# Impact of a pneumatic surgical robot with haptic feedback function on surgical manipulation

**DOI:** 10.1038/s41598-023-49876-7

**Published:** 2023-12-18

**Authors:** Yuichiro Ueda, So Miyahara, Keita Tokuishi, Hiroyasu Nakajima, Ryuichi Waseda, Takeshi Shiraishi, Toshihiko Sato

**Affiliations:** Department of General Thoracic Surgery, Breast and Pediatric Surgery, Fukuoka University School of Medicine, 7-45-1 Nanakuma, Jonan-ku, Fukuoka 814-0180 Japan

**Keywords:** Engineering, Physics

## Abstract

Although robotic-assisted surgery has the advantages of low patient burden and high precision without unsteady hand movements, the lack of tactile sensations may result in unexpected iatrogenic organ damage. The Saroa (Riverfield Inc., Tokyo, Japan) is a pneumatically driven robot that provides real-time haptic feedback to the surgeon. Using the Saroa robot, six examinees performed puffed rice transfer and four of them performed pig lung resection tasks with the feedback function turned on and off. The puffed rice transfer task consisted of transferring 20 grains of puffed rice from the left to the right compartment in the training box. The mean grasping forces during the puffed rice transfer task with the haptic feedback function turned off and on were 2.14 N and 0.63 N, respectively (*P* = 0.003). The mean grasping forces during the pig lung resection task were lower with the feedback turned on than turned off. The force that the forceps exerted on the grasping object was weaker in both tasks when the haptic feedback function was turned on, suggesting that the feedback function allows gentler handling of tissues, improving patient safety during robotic surgery.

## Introduction

Since the launch of the da Vinci® Surgical System (Intuitive Surgical, Sunnyvale, CA, USA) in 1999, robotic-assisted surgery has been widely performed worldwide. More than 10 million operations have been reported to date, and this number is expected to rise. Because robotic-assisted surgery uses articulated arms and non-swaying mechanisms, it is more precise than operations performed by human hands alone^1,2^. For example, a meta-analysis has shown that robot-assisted thoracic surgery (RATS) is a feasible and safe technique, with superior short-term and long-term outcomes compared with video-assisted thoracic surgery (VATS)^3^.

One important drawback of robotic-assisted surgery and other types of minimally invasive surgery (MIS) is the absence of tactile sensations, defined as a sense of touch caused by pressure or vibrations applied to the body. In contrast to open abdominal and thoracic surgery, during which surgeons can directly grasp organs with their hands, it is impossible for surgeons to experience full tactile sensations during abdominal and thoracic MIS. The force exerted on the tissue during MIS is based only on visually obtained information, resulting in the possibility of unexpected organ damage, particularly in thoracic surgery, as lung tissue is soft and can be easily damaged during grasping and deployment^4,5^. Incorporating a haptic feedback system into MIS tools is therefore important^6,7^, such that the use of surgical robots with a feedback mechanism can enhance surgical safety^8–13^. The definition of haptic feedback is to feed back the haptic force to the surgeon by reproducing the pressure applied to the forceps in real time. For this purpose, it is necessary to measure the pressure applied to the forceps from the robotic arm, convert it into an electrical signal, transmit it, and then reproduce the pressure applied the forceps at the controller side.

**Figure 1:**
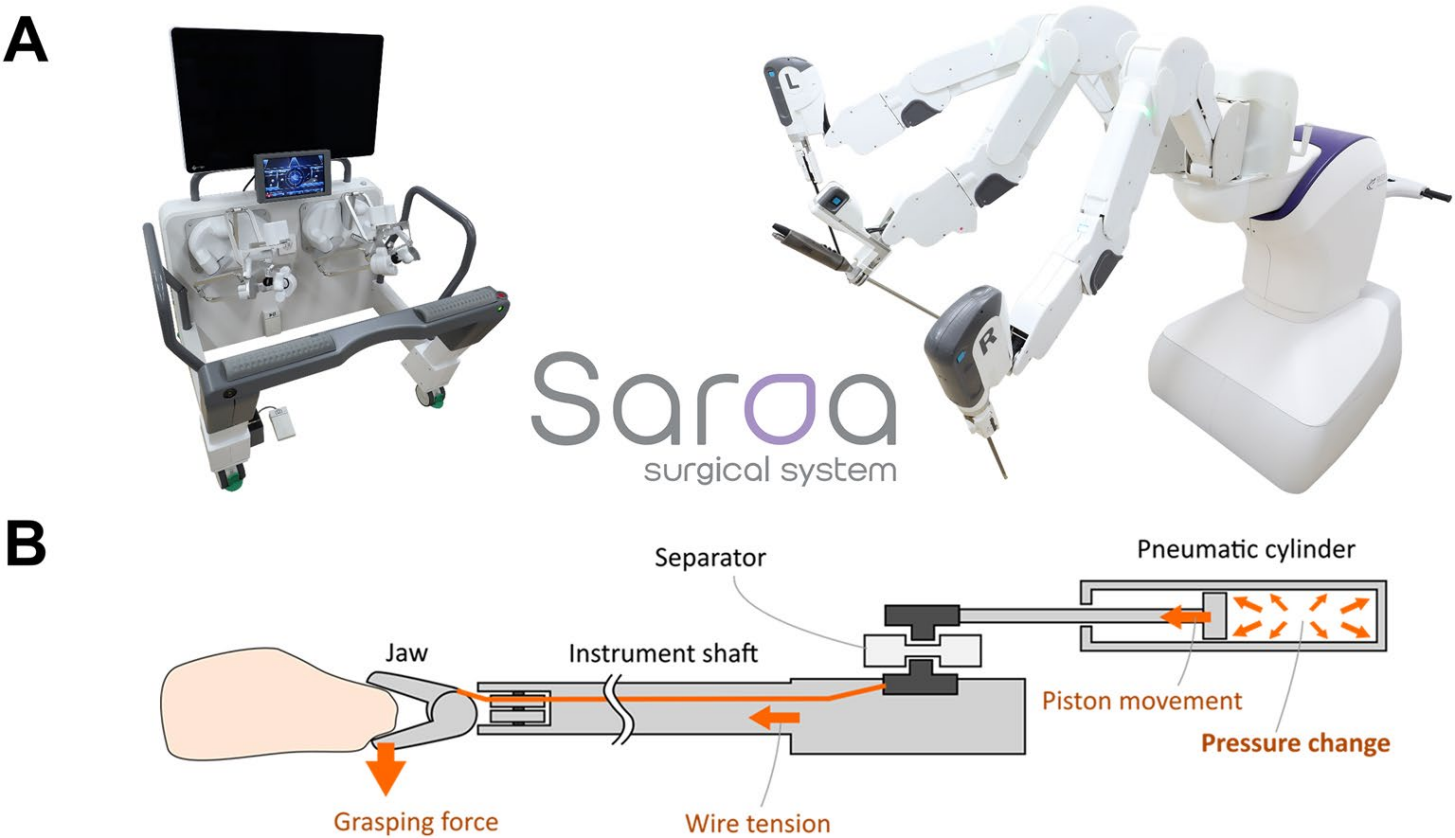
(**A**) Illustration of a “Saroa” surgical robot equipped with a haptic feedback mechanism. (**B**) Illustration of the mechanism of a haptic feedback.

The Saroa (Riverfield Inc., Tokyo, Japan) (Fig. 1A) is a pneumatically driven robot that provides real-time haptic feedback to the surgeon. In Saroa, the pneumatic cylinders implemented in the robotic arms drive the wires, that are contained in instruments and connected to the grasping jaw at the tip of instruments. The grasping force generated at the jaw is created by pressure in the cylinders. The pressure is continuously observed, and the measured value are translated to electrical signals. These electrical signals are sent to the hand controller, and based on that information, the force is reproduced and fed back in real time (Fig. 1B). Saroa can correctly estimate the grasping forces with less than 20% errors of the maximum output forces. The practical sensitivities of the external force estimation are better than 0.5 N. The force that the forceps exerted on the grasping object can be limited to 6 N. This means that the maximum force applied to the grasping object is much smaller than that of current surgical robots.

The usefulness of haptic feedback in robot-assisted surgery has been discussed in previous studies, but the experimental settings used in those studies were not ready for practical uses. This is because previous studies had experimented with forceps with small sensors attached externally, or with only the forceps portion of the robot, rather than the entire robot. Unlike those, Saroa has overcome issues such as measurement accuracy, sterility, biocompatibility, and cost-effectiveness, and can withstand clinical application. There has been no research on Haptic feedback using such a robot that can be applied in actual clinical practice. The haptic feedback function is expected to reduce tissue damage and the burden on the surgeon. This study evaluated the efficacy of haptic feedback function during surgery by measuring the amount of force applied to the forceps in real time.

## Materials and methods

**Figure 2:**
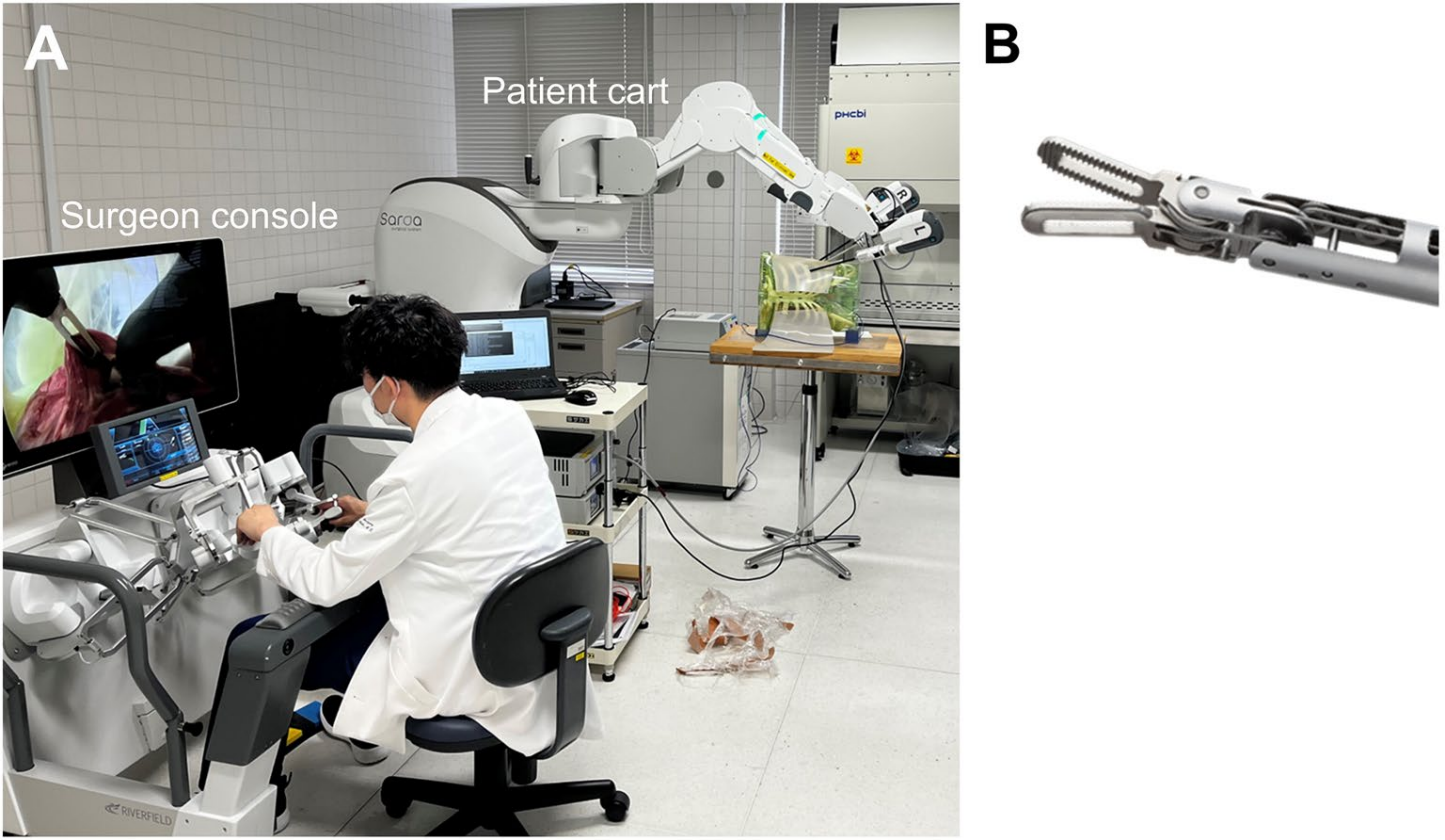
(**A**) Experimental view of puffed rice transfer and pig lung dissection tasks. (**B**) Fenestrated forceps of the Saroa surgical robot.

The study was conducted in accordance with the ethical standards of the Helsinki Declaration (1975), and was approved by the ethics committee of Fukuoka University Hospital (approval no. U23-06–008). Informed consent to participate in this study and publish identifying information and images were obtained from all participants. The experimental protocol was approved by the Animal Experimental Committee of Fukuoka University (approval No.2214090). The Saroa robot was rolled in (Fig. 2A), and fenestrated forceps (Fig. 2B) were set up through trocars placed in a training box. A personal computer and an endoscopic image recorder were attached to measure and record grasping force logs. To compare the degree of damage to objects grasped with forceps, the surface of a monofilament suture (4–0 proline™, ETHICON, USA) was ligated five times with da Vinci and Saroa (Video 1), with haptic feedback on and turned off, and assessed by scanning electron microscopy. Six physicians participated in the puffed rice transfer task and four of them in the pig lung dissection task.

### Puffed rice transfer task

**Figure 3:**
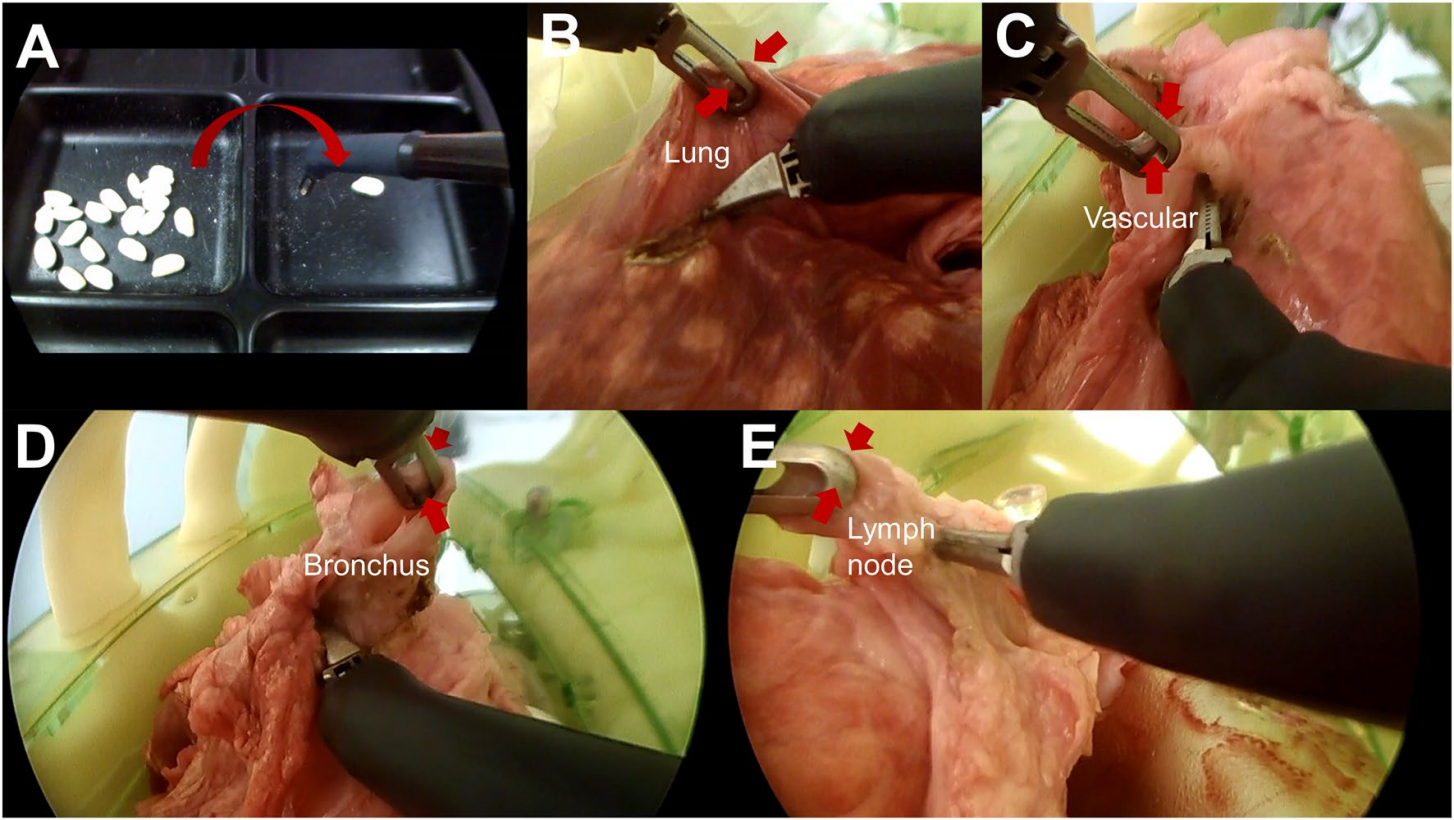
The arrows showing the movement of the forceps. (**A**) Illustration of the puffed rice transfer task in the training box. (**B**) Experimental view of interlobar dissection of the pig lung. (**C**) Experimental view of vascular dissection of the pig lung. (**D**) Experimental view of bronchial dissection of the pig lung. (**E**) Experimental view of lymph node resection of the pig lung.

Twenty grains of puffed rice (Hanahana Ltd., Hiroshima, Japan) were placed in a tray consisting of two compartments in the training box (Fig. 3A). We tested with various substances—for example, gummies, candies, and beads. Grain of puffed rice is a traditional Japanese snack made from rice, and although it has a certain degree of hardness, it is quite fragile. We chose to use this type of confectionery because of its superiority in evaluating the feedback function on and off. The participant used their dominant arm to transfer the 20 grains from the left compartment to the right compartment as quickly as possible (Video 2). The haptic feedback function was turned on and off blindly, and the amount of force per unit time (0.01 s) applied to the forceps was measured and recorded in real time. The time to task completion was also measured.

### Pig lung dissection task

Excised porcine lungs were subjected to interlobar dissection (Fig. 3B), vascular dissection (Fig. 3C), bronchial dissection (Fig. 3D), and lymph node resection (Fig. 3E). The force applied to the forceps during each of these tasks was measured with the haptic feedback function turned on and off blindly during the performance of each and recorded in real time.

### Analysis and evaluation

Any force > 0.1 N applied to the forceps with the feedback mechanism turned on and off was logged. The data were graphed, and the mean (to eliminate artifacts), integral value, and variance of force per unit time were measured and analyzed. In the puffed rice transfer task, the time until the end of the task was recorded. Differences in the amount of force exerted on the forceps were analyzed, including the dependence of this difference in force on the subject and type of task, its effect on the haptic feedback mechanism, and its efficacy in surgery. Means of continuous variables, such as grasping force, were compared by t- tests. All statistical analyses were performed with EZR (Saitama Medical Center, Jichi Medical University, Saitama, Japan) software, a graphical user interface for R (The R Foundation for Statistical Computing, Vienna, Austria). More precisely, EZR is a modified version of R commander designed to add statistical functions frequently used in biostatistics^14^.

## Results

### Scanning electron microscope findings

**Figure 4:**
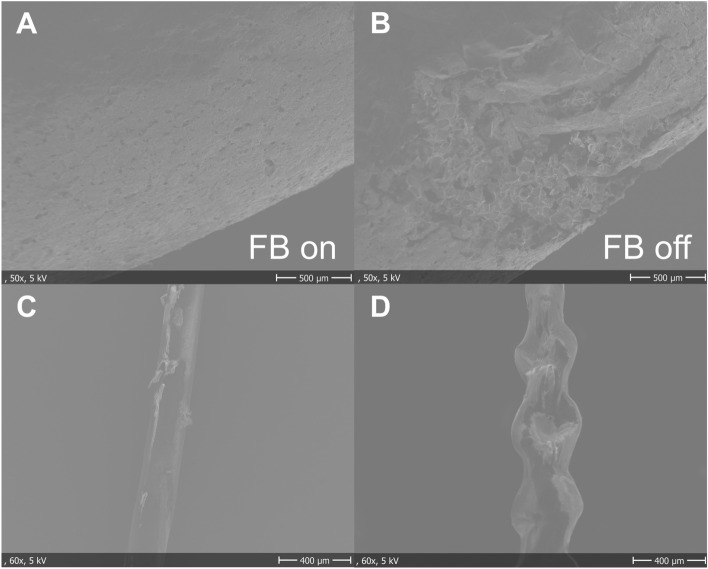
Scanning electron microscopy of puffed rice surfaces with a haptic feedback function turned on (**A**) and off (**B**). Scanning electron microscopy of monofilament sutures using a Saroa (**C**) and a da Vinci (**D**) robotic system.

Electron microscopic images of the puffed rice grains were analyzed. The degree of damage to the puffed rice surfaces varied, making quantitative evaluation difficult. However, when the haptic feedback function was on, there was no surface damage at all, and when it was off, most surfaces had forceps marks. Damage to the surfaces of puffed rice grains was lower with the feedback function turned on than turned off (Fig. 4A, B). The damage to the monofilament suture using a Saroa (Fig. 4C) was less than using a da Vinci (Fig. 4D).

### Puffed rice transfer task

**Figure 5:**
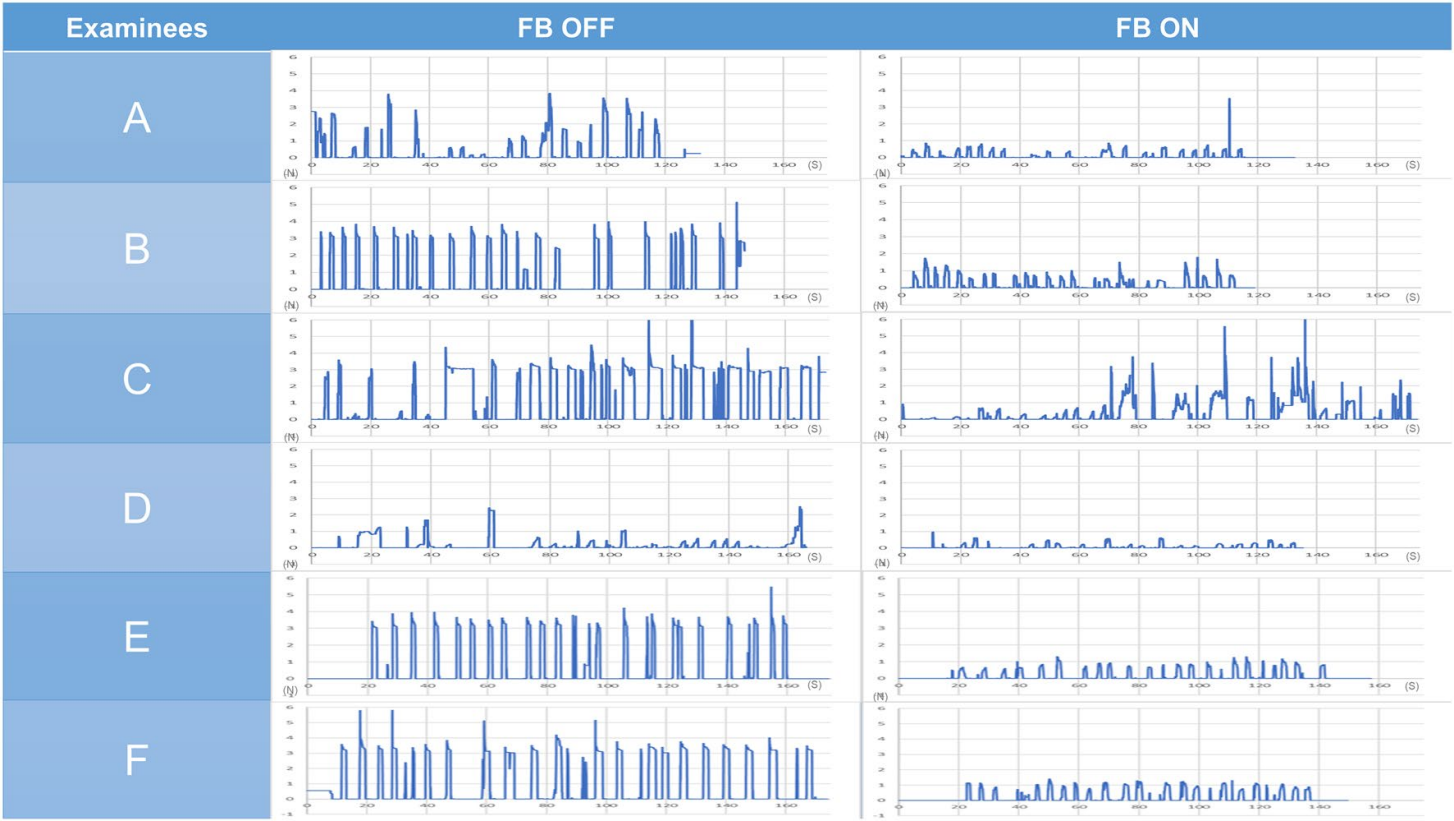
Graph showing changes in the amount of force applied to the forceps in the puffed rice transfer task.

**Figure 6:**
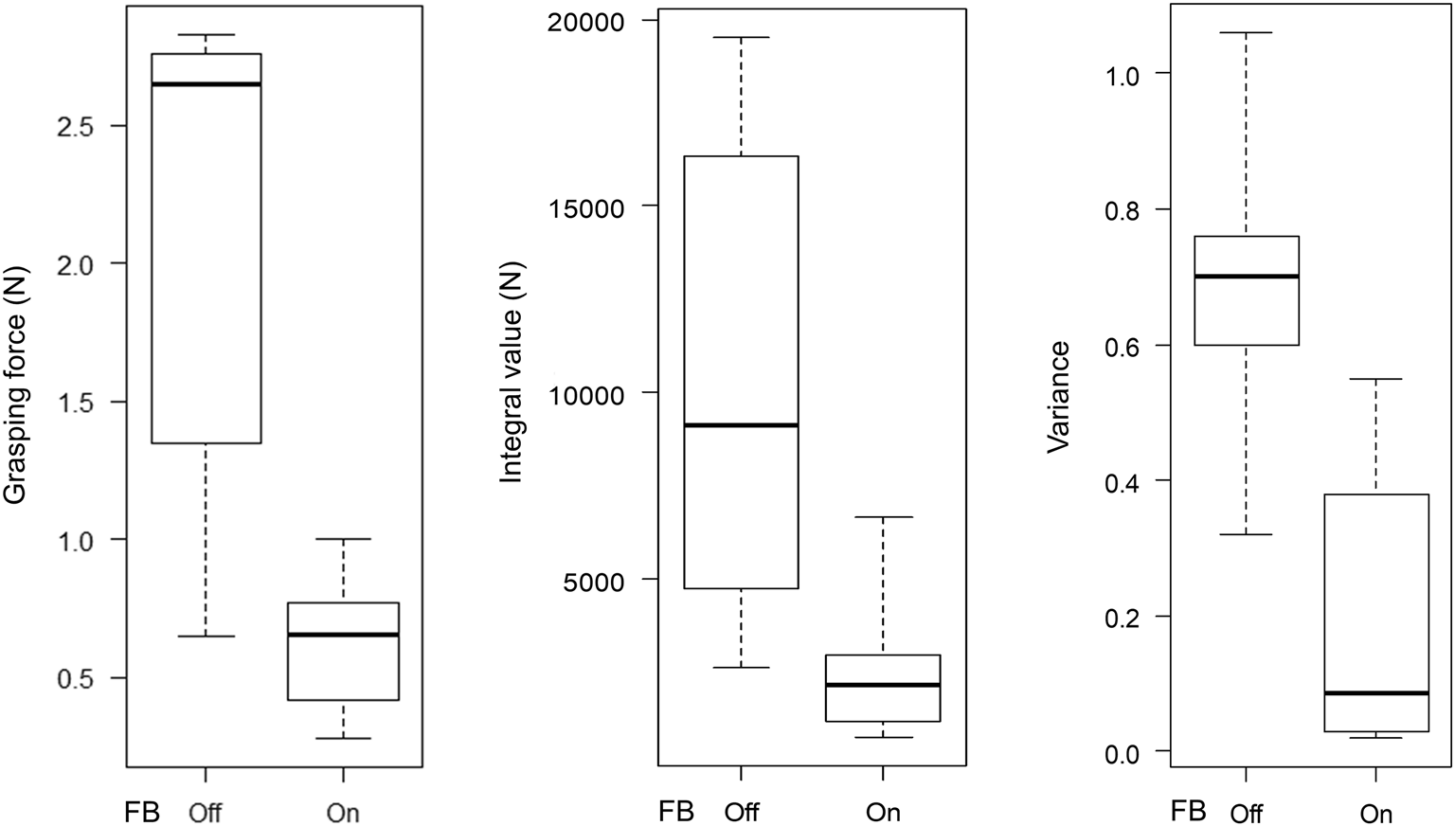
Boxplot showing grasping force, integral value, and variance in the puffed rice transfer task comparison with a haptic feedback function on and off.

The mean times to task completion were 153 s (range 118–172 s) with the haptic feedback function turned off and 127.8 s (range, 113–168 s) with haptic feedback function turned on (*P* = 0.07). The surgeon’s mean grasping force with the feedback turned off and on was 2.14 N (range 0.65–2.83 N) and 0.63 N (range 0.28–1.00 N) (*P* = 0.003), respectively; the mean integral value was 10,253 (range 2630–19,523) and 2648 (range 761–6656) (*P* = 0.02), respectively; and the mean variance was 0.69 (range 0.32–1.06) and 0.19 (range 0.02–0.55) (*P* = 0.003), respectively (Table 1, Figs. 5, 6).

**Table 1:** Outcomes of puffed rice transfer tasks performed by six examinees using the saroa system with the feedback function turned on and off.

Examinees	FB	Mean grasping force (N)	Variance	Integral force (N)	Time to task completion (s)
A	Off	1.35	0.76	4766	118
	On	0.42	0.07	1190	115
B	Off	2.83	0.70	19,523	172
	On	1.00	0.55	6656	168
C	Off	0.65	0.32	2630	169
	On	0.28	0.02	761	120
D	Off	2.74	0.70	8662	143
	On	0.64	0.10	2137	113
E	Off	2.56	1.06	16,354	170
	On	0.77	0.38	2949	119
F	Off	2.76	0.60	9581	146
	On	0.67	0.03	2195	132

*FB* Feedback, *s* second.

### Pig lung dissection task

**Figure 7:**
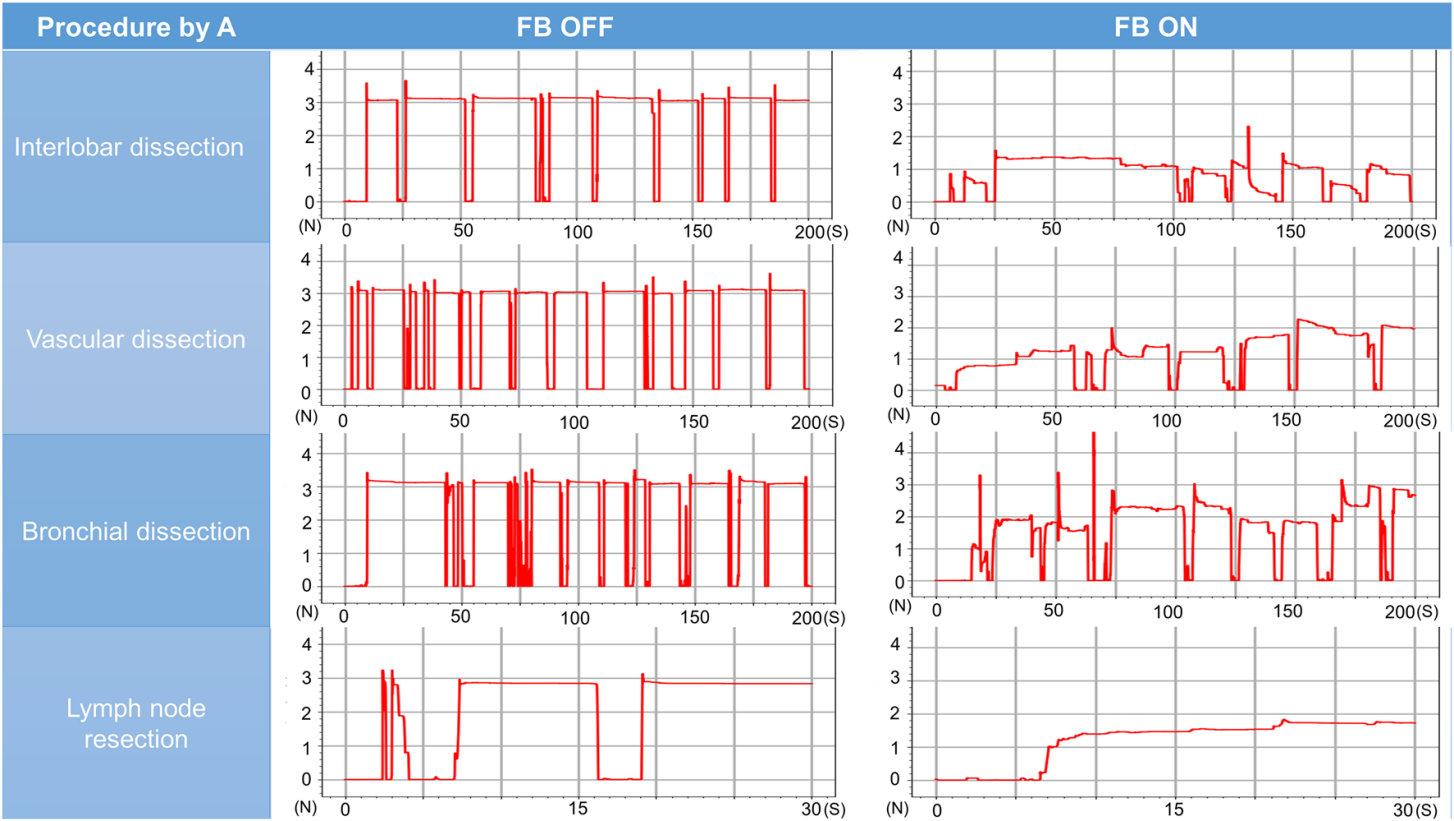
Changes in the amount of force applied to the forceps in pig lung dissection tasks performed by each of the four examinees.

**Figure 8:**
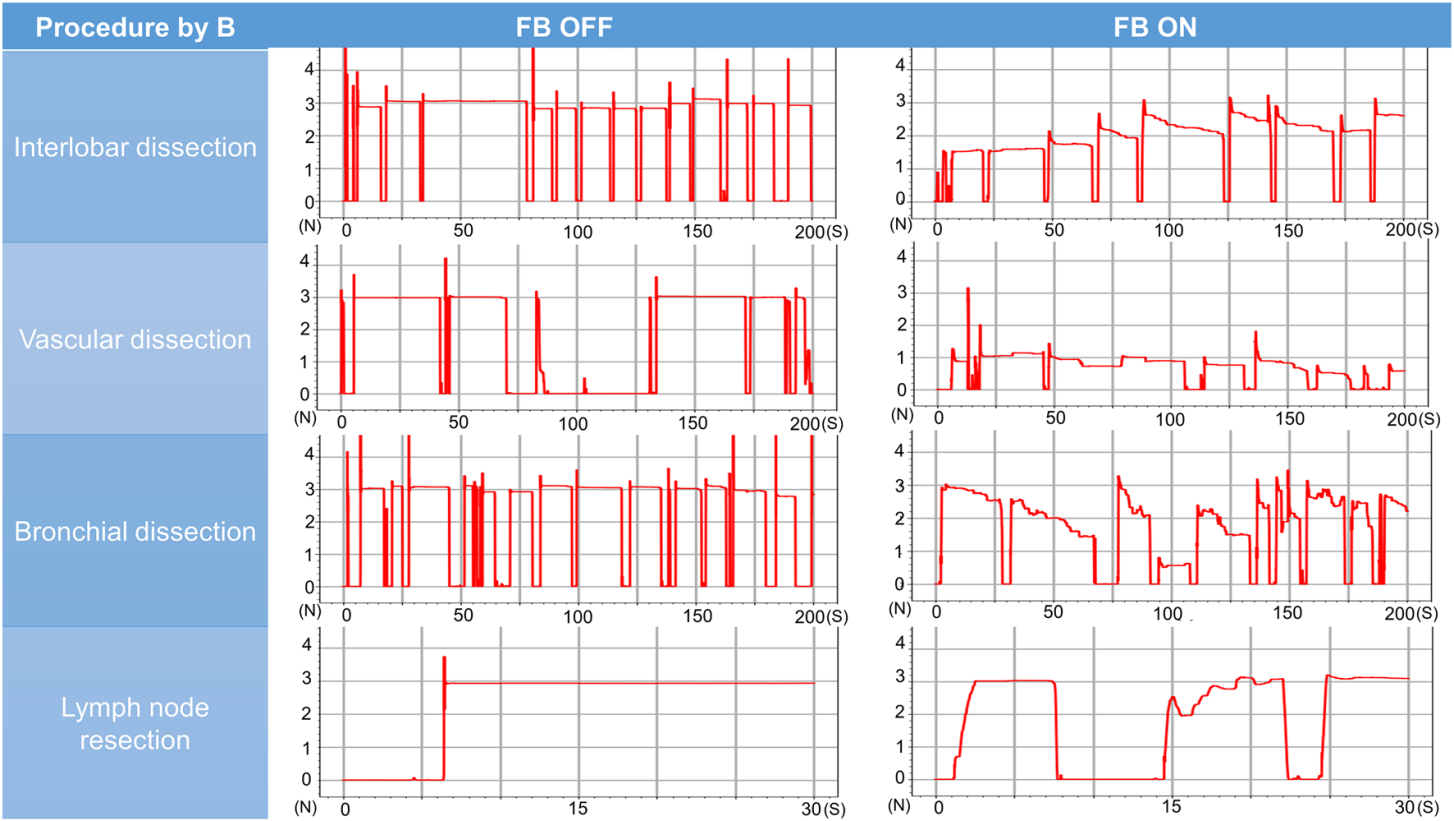
Changes in the amount of force applied to the forceps in pig lung dissection tasks performed by each of the four examinees.

**Figure 9:**
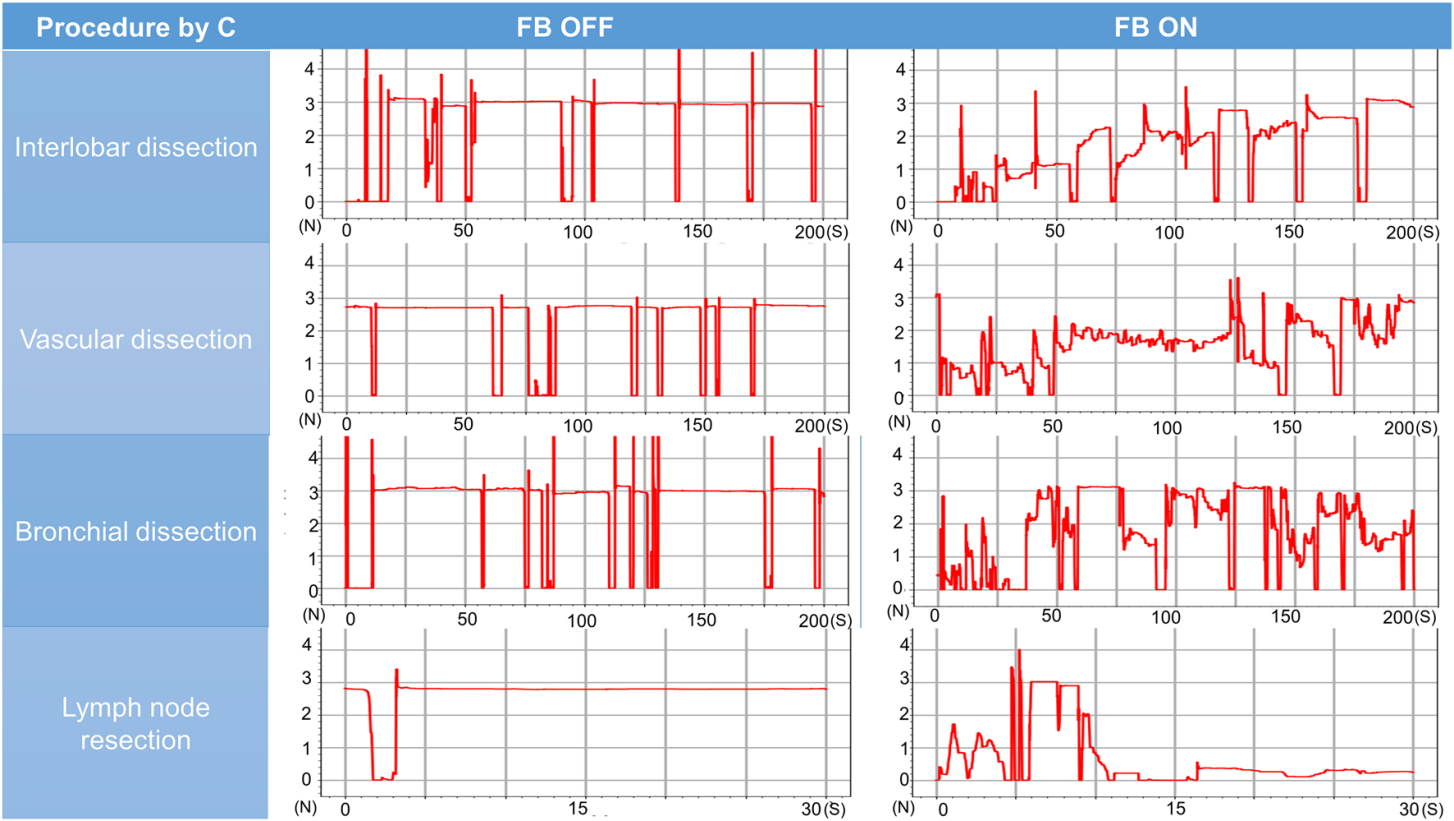
Changes in the amount of force applied to the forceps in pig lung dissection tasks performed by each of the four examinees.

**Figure 10:**
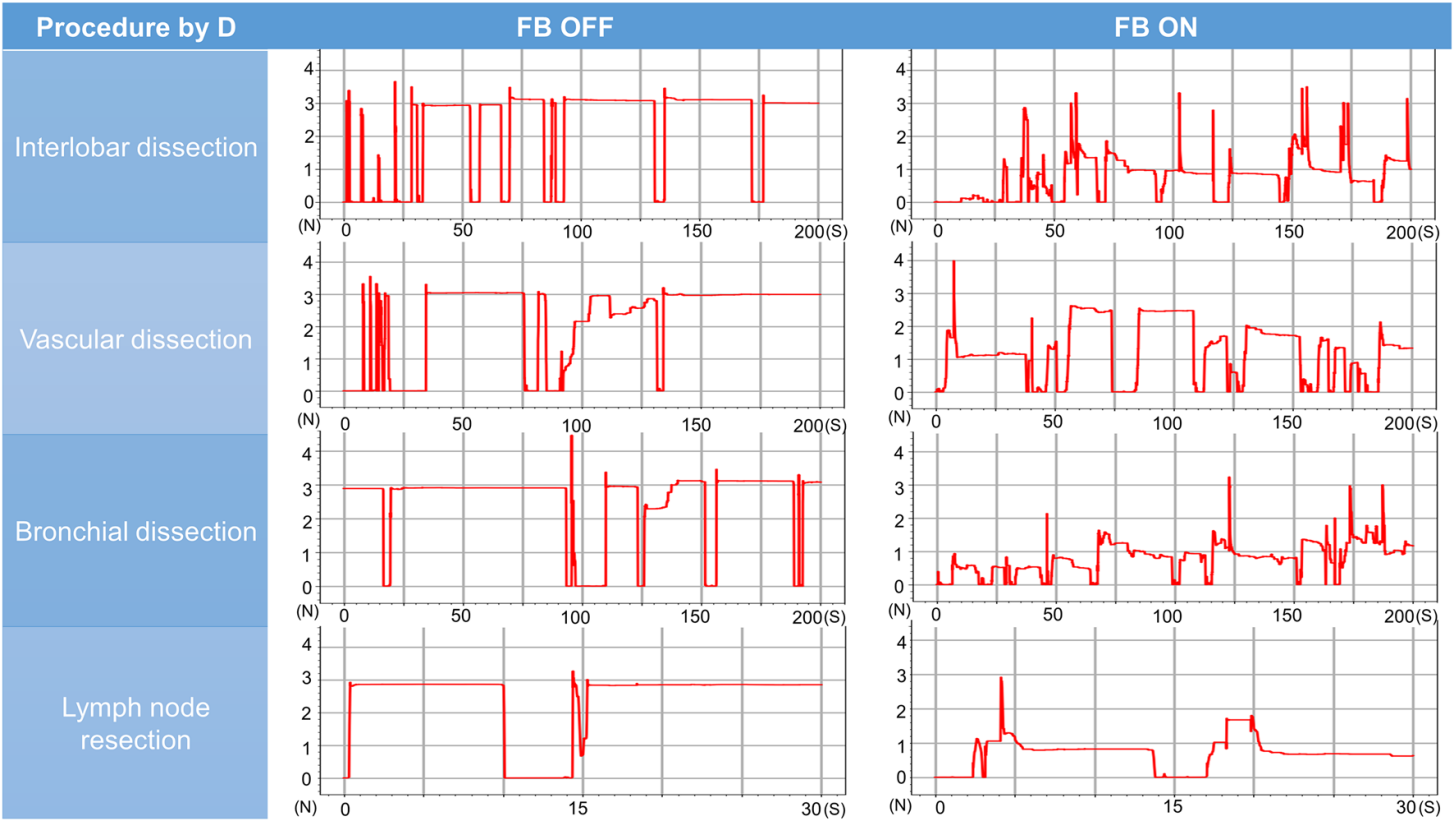
Changes in the amount of force applied to the forceps in pig lung dissection tasks performed by each of the four examinees.

**Figure 11:**
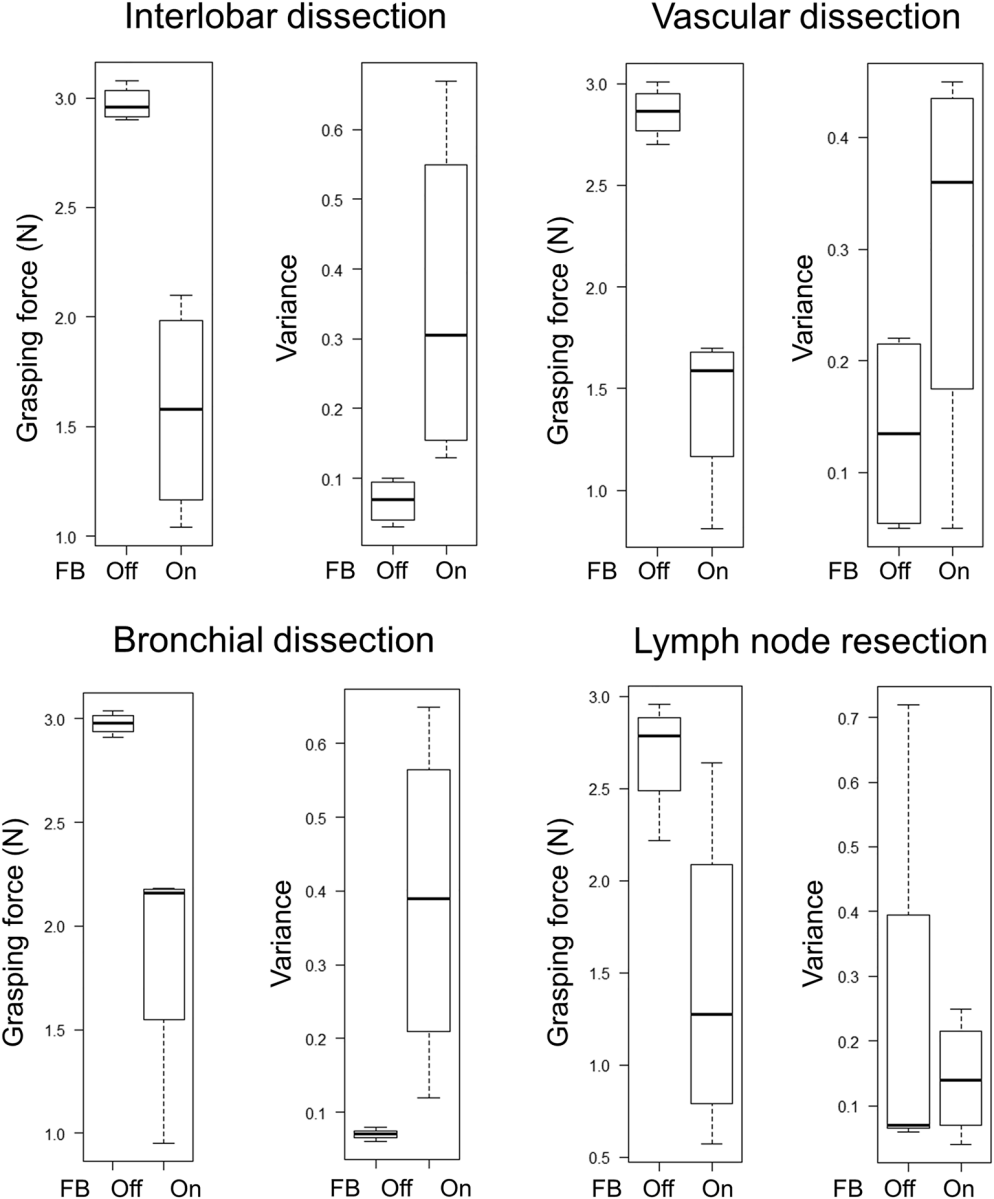
Boxplot showing grasping force, and variance in the pig lung dissection task comparison with a haptic feedback function on and off.

The surgeon’s mean grasping force during the pig lung resection task was lower with feedback turned off than turned on for interlobar dissection (2.98 N [range 2.90–3.08 N] vs. 1.58 N [range, 1.04–2.10 N], *P* = 0.01), vascular dissection (2.86 N [range, 2.70–3.01 N] vs. 1.42 N [0.81–1.70 N], *P* = 0.004), bronchial dissection (2.98N [range, 2.91–3.04 N] vs. 1.86 N [range, 0.95–2.18 N], *P* = 0.04), and lymph node resection (2.69 N [range, 2.22–2.96 N] vs 1.44 N [range, 0.57–2.64], *P* = 0.06). The mean variances differed with feedback turned off and turned on for interlobar dissection (0.07 [range, 0.03–0.10] vs. 0.35 [range, 0.13–0.67], *P* = 0.11), vascular dissection (0.14 [range, 0.05–0.22] vs. 0.31 [range, 0.05–0.45], *P* = 0.16), bronchial dissection (0.07 [range, 0.06–0.08] vs. 0.39 [range, 0.12–0.65], *P* = 0.07), and lymph node resection (0.14 [range, 0.04–0.25] vs. 0.23 [range, 0.06–0.72], *P* = 0.64) (Table 2, Figs. 7, 8, 9, 10, 11). Comparison of outcomes of puffed rice transfer and pig lung dissection tasks is shown in Table 3.

**Table 2:** Outcomes of pig lung dissection tasks performed by four examinees using the Saroa system with the feedback function turned on and off.

Task	Examinees	FB	Mean grasping force (N)	Variance
Interlobar dissection	A	Off	3.08	0.03
		On	1.04	0.13
	B	Off	2.90	0.10
		On	1.87	0.67
	C	Off	2.99	0.09
		On	1.29	0.43
	D	Off	2.93	0.05
		On	2.10	0.18
Vascular dissection	A	Off	3.01	0.06
		On	1.66	0.30
	B	Off	2.70	0.05
		On	1.70	0.42
	C	Off	2.84	0.21
		On	1.52	0.45
	D	Off	2.89	0.22
		On	0.81	0.05
Bronchial dissection	A	Off	3.04	0.06
		On	2.15	0.30
	B	Off	2.99	0.07
		On	2.17	0.65
	C	Off	2.91	0.07
		On	0.95	0.12
	D	Off	2.97	0.08
		On	2.18	0.48
Lymph node resection	A	Off	2.22	0.72
		On	1.54	0.04
	B	Off	2.76	0.07
		On	0.57	0.25
	C	Off	2.81	0.06
		On	1.01	0.10
	D	Off	2.96	0.07
		On	2.64	0.18

*FB* Feedback.

**Table 3:** Comparison of outcomes of puffed rice transfer and pig lung dissection tasks with and without the feedback system.

Task									
Puffed rice transfer	FB	Surgeon's mean Grasping Force (N)	*P*-value	Mean Variance	*P*-value	Mean integral force (N)	*P*-value	Mean time to task completion (s)	*P*-value
	Off	2.14	0.003	0.69	0.004	10,252.7	0.02	153	0.07
	On	0.63		0.19		2648		127.8	
Pig lung dissection									
Interlobar dissection	Off	2.98	0.01	0.07	0.11				
	On	1.58		0.35					
Vascular dissection	Off	2.86	0.004	0.14	0.16				
	On	1.42		0.31					
Bronchial dissection	Off	2.98	0.04	0.07	0.07				
	On	1.86		0.39					
Lymph node resection	Off	2.69	0.06	0.14	0.64				
	On	1.44		0.23					

*FB* Feedback; *s* second.

## Discussion

Haptic feedback has shown efficacy in estimating forceps force in patients undergoing specular surgery. For example, measuring and providing feedback of the force applied to the shaft of robotic forceps was found to suppress variations in tension during suturing^15^. In addition, simultaneous visual and haptic feedback information to the forceps was shown to improve the evaluation of tissue stiffness^8^. Although the haptic feedback function has been shown to improve surgical efficiency and safety, it has not been implemented to date^16^. There are two major approaches to realize Haptic feedback, one is to embed a small pressure sensor directly into the forceps tip, and the other is to measure the pressure applied to the entire forceps at the robotic arm side.

The former method is difficult to develop to a practical extent because it requires miniaturization, which reduces measurement accuracy, has significant sterilization and biocompatibility issues, and is not cost-effective. In the latter method, since there are no extra accessories for the forceps, issues of miniaturization, sterilization, and biocompatibility do not arise, and the forceps are not disposable, making it cost-effective. In the case of pneumatically driven robots like Saroa, the latter method can be used because it is easy to measure the pressure applied to the forceps. On the other hand, standard electrically driven robots cannot use the latter method. Because reduction ratio of electrically driven robots is high and force transmission is lost, making it difficult to measure force with high accuracy, so they must use a small sensor embedded in the forceps tip. This makes Saroa the first clinically appliable surgical robot with a haptic feedback. The Saroa device is pneumatically driven, making it more compact, lighter in weight, and less costly than the da Vinci surgical system, which is driven electrically. To our knowledge, the Saroa device is the first surgical robot for clinical applications equipped with a haptic feedback mechanism.

The present study assessed the ability of the Saroa device to perform two tasks: one involving a simple task, the transfer of puffed rice grains, and the other involving a more complex task, the resection of pig lungs. Although time to completion of puffed rice grain transfer did not differ when the feedback function was turned on or off, there were significant differences in mean grasping force and integral value. Scanning electron microscopy showed that the puffed rice surface was affected by forceps grasping when feedback was turned off but not when it was turned on. Thus, puffed rice grains were transferred with lower grasping force when feedback was turned on. Variances were generally lower when feedback was turned on, indicating that when feedback was turned on, the examinees were grasping and transferring puffed rice grains with a constant grasping force without task awareness.

Pig lung resection involved four procedures during which the grasping force was measured. Lung injury may occur during interlobar dissection, especially during traction; the lungs of patients with underlying diseases, such as emphysema or interstitial pneumonia, are particularly vulnerable to injury. Grasping traction force on the lungs was lower when feedback was turned on, indicating that interlobar dissection was possible with a low grasping force without lung injury. Vascular dissection involves grasping thin-membraned structures such as the vascular sheath and surrounding tissue, whereas dissection of the peribronchial area requires grasping of hard tissue such as bronchi. During both procedures, the tissues and membranes were more likely to be grasped with lower force when feedback was turned on than turned off, indicating that manipulation was gentler when feedback was turned on. Lymph node resection was also performed with weaker force when feedback was turned on than off, although the difference was not statistically significant. Although variances did not differ significantly, variances in interlobar and bronchial dissection were lower, whereas variances in vascular dissection and lymph node extraction were higher, when feedback was turned on. These findings suggested that the same area is often grasped and pulled in the lungs or bronchi, and that turning on the haptic feedback function results in application of a constant force. During vascular dissection and lymph node removal, however, the grasping site changes; the examinees may have unconsciously adjusted the amount of force while grasping when the haptic feedback function was turned on.

This study had several limitations. First, the number of examinees was small; however, all six showed the same tendencies when the haptic feedback function was turned on or off, a result likely to occur in a larger sized sample. Second, tissue damage was not evaluated histologically. Further studies are required to evaluate and confirm that histologic damage is reduced by turning on the feedback function.

In conclusion, this study showed that the haptic feedback function of the Saroa robot reduced the forceps’ grasping force during puffed rice transfer and pig lung dissection. This robotic system may contribute to improvements in surgical safety by facilitating precise procedures that are gentler on the tissue.

### Supplementary Information

Supplementary Legends.

Supplementary Video S1.

Supplementary Video S2.

Supplementary Video S3.

## Data Availability

The datasets used and/or analysed during the current study available from the corresponding author on reasonable request.
