# Supplementary material for: Impact of a pneumatic surgical robot with haptic feedback function on surgical manipulation
Source: Sci Rep. 2023 Dec 18;13:22615. doi: 10.1038/s41598-023-49876-7 (PMC10730604; doi:10.1038/s41598-023-49876-7)
Supplement: Supplementary file 1 — Supplementary Legends. [file 41598_2023_49876_MOESM1_ESM.docx]

**Video 1.**

Intraoperative view of suturing the right lower bronchial stump with da Vinci using 4-0 proline and experimental view of ligating five times with the Saroa.

**Video 2.**

Experimental view of comparison with haptic feedback function turned on and off in the puffed rice transfer task.

**Video 3.**

Experimental view of comparison with haptic feedback function turned on and off in the pig lung dissection task.
